# Health-related quality of life in colorectal cancer survivors: are there differences between sporadic and hereditary patients?

**DOI:** 10.1186/s41687-018-0047-4

**Published:** 2018-05-01

**Authors:** Allison M. Burton-Chase, Wendy M. Parker, Kirsten M. Donato, Shannon McCormick, Ellen R. Gritz, Christopher I. Amos, Karen H. Lu, Patrick M. Lynch, Miguel A. Rodriguez-Bigas, Y. Nancy You, Susan K. Peterson

**Affiliations:** 1 Department of Population Health Sciences, Albany College of Pharmacy and Health Sciences, 106 New Scotland Avenue, Albany, NY 12208 USA; 2 Department of Behavioral Science, The University of Texas MD Anderson Cancer Center, Houston, TX USA; 3 Department of Community and Family Medicine, Dartmouth College, Hanover, NH USA; 4 Department of Gynecologic Oncology & Reproductive Medicine, The University of Texas MD Anderson Cancer Center, Houston, TX USA; 5 Department of Gastroenterology, Hepatology, & Nutrition, The University of Texas MD Anderson Cancer Center, Houston, TX USA; 6 Department of Surgical Oncology, The University of Texas MD Anderson Cancer Center, Houston, TX USA

## Abstract

**Purpose:**

To compare health-related quality of life (HRQoL) in colorectal cancer (CRC) survivors with sporadic CRC to those with hereditary cancer, specifically Lynch syndrome (LS).

**Methods:**

Participants completed a mailed self-administered questionnaire that assessed, among other things, demographics, clinical characteristics, and health-related quality of life. Using a case-case design, CRC survivors with LS or sporadic cancer were matched on age, sex, race/ethnicity, cancer stage, geography, and time since diagnosis. Participants were recruited from patient registries at The University of Texas MD Anderson Cancer Center (MD Anderson) (*n* = 33 LS; *n* = 75 sporadic) and through social media (*n* = 42 LS). The final sample included 71 LS and 74 sporadic CRC survivors.

**Results:**

For LS patients, the mean FACT-C HRQoL score was 84.8 (11.9) [Median = 86.0; Interquartile Range-17] compared to sporadic patients mean score of 85.8 (16.7) [Median = 92.0; Interquartile Range-21], which indicates high quality of life for both groups. LS patients and sporadic CRC patients had similar HRQoL mean scores across 7 different HRQoL metrics, with no significant differences between groups. Exploratory regression analyses indicate some differences in known predictors of HRQoL by group despite no bivariate differences.

**Conclusions:**

HRQoL is an important component of survivorship in CRC patients. Given the clinical distinctions between LS and sporadic patients, we expected to find significant differences between these patients. However, the patients’ experiences/quality of life does not appear to illustrate such a clear dissimilarity within CRC survivors. Given the limited data in this area, larger studies, ideally with data obtained from multiple sites, is needed to better investigate the alignment between clinical determination and patient experience as well as to explore the relationship between HRQOL, treatment regimens, and health outcomes.

## Introduction

Health-related quality of life (HRQoL) is considered an important factor to assess in all colorectal cancer survivors (CRC), particularly because it has been shown to have an impact on screening and surveillance decisions [1]. Many complex factors have been found to contribute to HRQoL in colorectal cancer (CRC) survivors, including gender, age, income, education level, social network, body mass index, and high number of comorbidities [1]. Marventano et al. in a 2013 study found that the presence of comorbid conditions, such as diarrhea, pain, incontinence, and fatigue, may be attributable to the patient’s past history of CRC [2]. These conditions directly limited a patient’s ability to participate in daily activities and resulted in a higher prevalence of distress, depression, and anxiety compared to the general population [2]. Clinical studies have increasingly incorporated HRQoL as an important endpoint to understanding patient care experiences. The American Society of Clinical Oncology, in 1996, identified HRQoL as one of the important elements to be measured in cancer patients, as its effect on symptom severity was undervalued in older studies [3]. Patient and provider decisions regarding screening, surveillance, and treatment, as well as decisions about genetic testing, can be significantly impacted by HRQoL; whether a patient has sporadic or hereditary CRC also impacts these decisions. In the current study, we compared HRQoL in CRC survivors with Lynch syndrome (LS) to those with sporadic cancer, given that there are significant differences in screening and surveillance recommendations and cancer risks for these two survivor populations.

Approximately 70% of CRC cases are due to sporadic cancer [4]. LS accounts for approximately 3% of all CRC cases and is identified through clinical genetic counseling and testing, ideally initiated in individuals with cancer [5, 6]. Compared with the general population, individuals with LS have higher lifetime risks for several cancers; CRC risk ranges from 20 to 69% for men and 10–52% for women, with risks varying based on the mutated gene [7–11]. Women with LS have a 40–60% lifetime risk for developing endometrial cancer [6, 12–14]. Individuals with LS also are at increased risk for other cancers, including ovarian, stomach, small bowel, hepatobiliary tract, pancreatic, urinary tract, brain, and skin cancers [6, 12, 14]. As a result of these significantly elevated risks, individuals with LS are advised to follow high-risk screening and surveillance guidelines, which include: 1) annual or biennial colonoscopy initiated at age 20–25 years or 2–5 years younger than the earliest known case in the family; and, for women 2) annual endometrial screening initiated at age 30–35 years. Surveillance recommendations for other LS-related cancers are determined on a case-by-case basis taking family history and gene mutation into consideration, as there have been no reported benefits for these recommendations. In some cases prophylactic surgery also is recommended [6, 12, 15, 16].

HRQoL can be an important factor in prevention of disease, but patients and providers often have differing perspectives on the manner and timing of screening [1]. For CRC specifically, providers prefer colonoscopy as a screening tool because it allows for visualization of the entire bowel and easy removal of suspicious lesions as well as significant precancerous lesions. From a patient perspective, however, colonoscopy may be less acceptable, due to the required preparation procedure and worry regarding pain, discomfort, and complications. A recent study concluded that the recommended CRC screening protocol should be audited for quality and HRQoL should be assessed as a mechanism to promote adherence to screening [1].

The relationship between HRQoL and cancer treatment options has been assessed in prior research, however results have been mixed. Studies looking at the relationship between specific types of treatments, such as post-laparoscopic colectomy, pre-operative short-course radiotherapy treatment, and pre-operative long course chemo-radiotherapy, and QoL have reported divergent findings [17, 18]. For radio- and chemo-radiotherapy, one study found no differences in HRQoL between the treatment groups [18]. In a review article examining HRQoL in colectomy patients, HRQoL was low at baseline due to worry and distress attributed to the recent cancer diagnosis, but within one year patients had HRQoL scores comparable to the general population [17]. Both gender and age appear to impact QoL in this group of colectomy patients, with emotional function being more affected in women and sexual functioning being more affected in men [17]. Additionally, HRQoL scores tended to be higher in younger patients, which was attributed to having experienced a lower number and severity of complications in comparison to older patients [17]. Both of these studies focused on the general CRC patient population and we are unaware of any studies, to date, that have examined these factors in individuals with LS.

There is a small body of literature examining the relationship between HRQoL and genetic testing in the LS population. Specifically, HRQoL has been shown to have an impact on physician and patient decisions regarding LS genetic testing, with physicians acknowledging that patient worry can decrease referrals and uptake [19]. Overall, physicians report that their knowledge of and experience with LS and the importance of preventive health behaviors in this population are major factors in genetic testing referral [19]. Genetic testing also has been studied in individuals with LS to determine its psychological impact as well as its impact on screening behaviors. One study found that QoL did not differ significantly between individuals who tested negative for LS and those who tested positive [20]. As expected, individuals with a positive test result had higher perceived risk of developing CRC than those with a negative test result [20]. However, individuals who tested negative still reported feeling anxiety and they overestimated their risk for CRC, likely due to factors such as having a family history of CRC or prior experience with family or friends having had cancer [20]. Taken together, research with both sporadic and LS-associated CRC patients has shown that HRQoL and adjustment to genetic testing results is impacted by demographic factors, coping style, social support, family history, and prior cancer experience [1, 20]. Additionally, patients with higher perceived cancer risk and those with positive genetic test results are more likely to follow screening and surveillance recommendations [20, 21]. These findings indicate both that HRQoL is an important factor in genetic testing for LS and that, combined with patient’s having accurate knowledge about their cancer risks, HRQoL has an impact on uptake of appropriate screening and surveillance.

Multiple factors that contribute to HRQoL in various cancer patients with or without LS influence their choices regarding prevention, treatment, and genetic testing. Although there are now more data regarding HRQoL than previously, there is still a paucity of evidence in CRC patients. Few studies have compared HRQoL across different groups of cancer patients or used valid instruments to measure HRQoL [22]. To address this gap in the literature, we compared HRQoL between two groups of CRC survivors, those with and without LS. To our knowledge, there is no prior literature on this topic. It is important to understand HRQoL in CRC survivors, especially those with LS, to better understand how it contributes to screening and surveillance adherence, genetic counseling decisions, and treatment plans in this population [21]. Additionally, assessing differences between these two groups will provide insight into whether CRC survivors with LS require a more thorough assessment of HRQoL compared to sporadic CRC survivors.

## Methods

### Participants

This study was approved by the Institutional Review Board at the University of Texas MD Anderson Cancer Center (MD Anderson). Participants were CRC survivors with LS or sporadic cancer who were matched on age, sex, race/ethnicity, cancer stage, geography, and time since diagnosis using a LS case-sporadic case design. Survivors with LS were recruited from MD Anderson (*n* = 33) and through social media (*n* = 42) and had to have tested positive for a LS mutation. Sporadic CRC survivors were recruited from the tumor registry at MD Anderson (*n* = 75). All participants were 18 years of age or older and were able to read and speak English. CRC patients were limited to those with a diagnosis of CRC from 6 months to 5 years prior to enrolling in the study. LS participants recruited through MD Anderson were screened for eligibility using medical records and those recruited through social media were screened by self-report over the phone prior to enrolling in the study. Using information from their medical records, we excluded sporadic CRC patients with a personal or family history of familial adenomatous polyposis (FAP), inflammatory bowel disease, or those who had a first-degree relative with CRC.

### Data collection methods

Data were collected using a mailed, self-administered questionnaire. Eligible survivors received a packet containing an introductory letter, questionnaire, and a self-addressed stamped return envelope. Nonrespondents received an identical follow-up mailing at 3 weeks after the initial mailing and a follow-up reminder phone call at 6 weeks with the option to complete the questionnaire over the phone. Those who completed the questionnaire received a $10 gift card as compensation.

### Study measures

Demographic data were obtained through self-report; medical data were obtained through medical records and self-report. Depression was measured using the Center for Epidemiological Studies-Depression Scale (CESD-D) [23]. The Functional Assessment of Cancer Therapy for Patients with Colorectal Cancer (FACT-C), a cancer-specific measure associated with the Functional Assessment for Chronic Illness Therapy (FACIT), was used to assess health-related QOL [24–27]. The FACT-C contains four well-being subscales: physical, social/familial, emotional, and functional. The FACT-C has shown good internal consistency, reliability, and concurrent validity, as well as an ability to distinguish between groups based on functional status and extent of disease [24–26]. The FACT-C adds a fifth subscale that assesses colorectal-cancer specific quality of life [25, 26]. Additionally, overall HRQoL as well as colorectal cancer-specific HRQoL were assessed using these measures [24–26]. Overall HRQoL was assessed using the sum of the physical, social/familial, emotional, and functional subscale scores; colorectal-cancer specific HRQoL added the additional concerns subscale score to the original 4 subscale scores. For all HRQoL measures, higher scores indicate higher levels of functioning.

### Statistical analysis

LS and sporadic patients (*n* = 150) were analyzed separately and compared to each other on sociodemographic, health care utilization, and QoL measures using SAS version 9.4 © SAS Institute, Inc. to look for group differences. Small amounts of missing data were mean imputed except for outcome measures. The final sample size, after excluding those missing on HRQoL measures, was *n* = 71 (LS) and *n* = 74 (Sporadic). Given limited bivariate differences, exploratory regression analyses were completed via SAS to look at predictors of HRQoL and FACT-C metrics for LS and Sporadic CRC patients separately.

## Results

As expected, due to the recruitment method, there are no significant differences between LS and sporadic CRC survivors on demographics [Table 1]. There also are no differences on health care utilization. The one exception is treatment location at a comprehensive cancer center (CCC), which also is due to the recruitment method. Overall, the study population is around 50 years of age, slightly more likely to be female, married, White, currently employed, and has at least a college degree with few financial difficulties.

**Table 1:** Demographic characteristics of Lynch syndrome (LS) and sporadic participants [n (%)]

Characteristic		LS (*n* = 71)	Sporadic (*n* = 74)	*p*-value
Mean Age (SD)		52.3 (12.0)	54.9 (11.6)	0.18
Sex	Male	32 (45.1%)	33 (44.6%)	0.21
	Female	39 (54.9%)	41 (55.4%)	
Marital Status	Married	61 (85.9%)	56 (75.7%)	0.18
	Not Married	10 (14.1%)	18 (24.3%)	
Race	White	65 (91.6%)	70 (94.6%)	0.52
	Non White	6 (8.5%)	4 (5.4%)	
Child	Have Children	58 (81.7%)	66 (89.2%)	0.12
	No Children	13 (18.3%)	8 (10.8%)	
Work	Working Full or Part Time	46 (64.8%)	43 (58.1%)	0.41
	Not Working	25 (35.2%)	31 (41.9%)	
Education	Less than College	27 (38.0%)	35 (47.3%)	0.28
	College Degree	22 (31.0%)	25 (33.8%)	
	Post Graduate	22 (31.0%)	14 (18.9%)	
Financial Situation	Financial Difficulty	13 (18.3%)	11 (14.9%)	0.71
	No spare money	19 (26.8%)	24 (32.4%)	
	Can afford special things	39 (54.9%)	39 (52.7%)	
Location of Treatment	Comprehensive Cancer Center (CCC)	31 (43.7%)	74 (100%)	0.001***
	Non CCC	40 (56.3%)	0 (0%)	
Health Care Experiences (Number)	Doctor visits (past 6 months)	4.0 (3.8)	3.5 (2.8)	0.44

***p < .001 NOTE: Not all percentages and totals add up due to missing data

For the seven HRQoL measures, we found that both LS and sporadic CRC survivors score high on all measures and there are no significant differences between the two groups [Table 2]. Overall HRQoL (LS mean = 84.8; Sporadic mean = 85.8, with a maximum score of 108), which is a summary measure of the physical well-being (PWB), social/family well-being (SWB), emotional well-being (EWB) and functional well-being (FWB), demonstrates that both LS and sporadic patients generally feel a sense of happiness in their quality of life experiences.

**Table 2:** FACT-C Health-Related Quality of Life Mean Scores

Mean (SD)	LS (*n* = 71)	Sporadic (*n* = 74)	Maximum Score	*P*-value
FACT-G General Scores for Health Related Quality of Life *[Total FACT scores: PWB + SWB + EWB + FWB]*	84.8 (11.9)	85.8 (16.7)	108	0.68
Total FACT-C Scores for Health-Related Quality of Life Colorectal *[Total FACT scores plus Additional Concerns Questions: PWB + SWB + EWB + FWB + AC]*	105.8 (14.1)	106.3 (19.9)	134	0.85
Physical Well Being (PWB)	23.8 (4.1)	23.2 (4.9)	28	0.45
Social Family Well Being (SWB)	19.9 (5.4)	21.0 (5.5)	28	0.25
Emotional Well Being (EWB)	19.6 (3.1)	19.8 (4.1)	24	0.70
Functional Well Being (FWB)	21.5 (4.6)	21.7 (5.8)	28	0.74
Additional Concerns (AC)	21.0 (4.2)	20.5 (4.6)	28	0.56

Given the limited data available for CRC survivors, especially LS patients, we examined predictors for the HRQoL metrics, despite the lack the of group differences at the bivariate level. Exploratory regression results show limited predictors of HRQoL measures. For LS patients, treatment at a CCC is positively associated with higher HRQoL and an elevated CES-D score (indicating depressive symptoms) is associated with lower HRQoL. For sporadic CRC patients being married is positively associated with higher HRQoL and depressive symptoms (CES-D) is negatively associated with HRQoL. This pattern holds for nearly all subscales with minor variations (Supplemental tables available upon request).

## Discussion

While the clinical significance of a LS genetic test is well-documented in terms of cancer risk management, screening, and surveillance demands, there are limited data on the patient experience in terms of HRQoL for both LS and sporadic CRC survivors. To the best of our knowledge, this study is the first to compare HRQoL across these two groups. The findings of this study indicate that LS and sporadic patients may be more alike than they are different in terms of their emotional, functional, social, and physical well-being, as well as their overall HRQoL. Both groups scored highly on this measure, which could potentially indicate resilience in this population. Previous research has noted the importance of worry in a patient’s decision to adhere to screening and surveillance recommendations [1]. Given our findings that these two groups are similar on HRQoL, LS and sporadic CRC survivors may both need to be assessed for worry and provided similar encouragement by health care providers to adhere to recommended screening protocols. Additionally, our exploratory models assessing predictors of HRQoL indicated that depression has a negative impact on HRQoL for both sporadic and LS-associated CRC survivors. While we are cautious in making any recommendations based on this finding, this relationship should be explored in future studies. If confirmed, health care providers can assess depressive symptoms in their CRC survivors and target interventions to individuals displaying these symptoms, in order to increase HRQoL and, subsequently, screening and surveillance adherence.

In prior data comparing QoL between individuals who tested positive and those who tested negative for LS, results showed that there were no significant differences between the two groups [20]. However, patients who tested negative still reported feelings of anxiety and also overestimated their cancer risks [20]. Our finding that there were no differences between CRC survivors with and without LS extends the limited literature in this area and suggests that QoL and HRQoL may be more global measures that are not easily influenced by changes in clinical status. Given existing literature showing that HRQoL influences adherence to screening and surveillance as well as decisions regarding treatment and genetic testing, the conclusion from a recent study that HRQoL should be a standard assessment in clinical practice to improve the quality of care merits consideration by health care providers [1].

Our findings provide information that is relevant to both clinicians and researchers regarding to HRQoL in CRC survivors and contributes to the small body of literature that exists for both sporadic and hereditary CRC survivors. We recognize that our study is limited by the small sample sizes of both groups as well as the potential bias in recruitment from two different pools of LS patients. The sample size also limited our ability to draw conclusions from our exploratory models assessing predictors of HRQoL in these two groups. Additionally, the time since diagnosis ranged from 6 months to 5 years. While this allowed for us to assess a survivorship population, it also introduces variation in terms of stage of survivorship. Ideally, longitudinal data would be collected in this population. Recruitment for this hard-to-reach hereditary cancer population as well as case-case matching was painstakingly undertaken to ensure high quality data. This methodology, along with the selection of sensitive and disease-specific measures, are both strengths of the study.

HRQoL is associated with adherence and positive health behaviors in oncology patients. As a rare disease with significant clinical implications, understanding HRQoL in LS patients is imperative to support effective genetic testing, screening and surveillance, and treatment. Our findings suggest that HRQoL may be a global metric, which could make it a valuable patient assessment tool for all CRC survivors.
